# Anti‐cancer drugs targeting the NADH‐binding site of VDAC rewire channel electrophysiology and partially suppress cation selectivity

**DOI:** 10.1111/febs.70434

**Published:** 2026-02-09

**Authors:** Stefano Conti‐Nibali, Giuseppe Battiato, Salvatore Antonio Maria Cubisino, Cristina Arrigoni, Marco Lolicato, Simona Reina, Vito De Pinto

**Affiliations:** ^1^ Department of Biomedical and Biotechnological Sciences University of Catania Italy; ^2^ Department of Molecular Medicine University of Pavia Italy

**Keywords:** anti‐cancer compounds, cation selectivity, mitochondria, NADH‐binding pocket, VDAC gating

## Abstract

Located at the crossroads between mitochondria and cytosol, VDAC1 (Voltage‐Dependent Anion Selective Channel isoform 1) serves as the chief actor in the regulation of cell metabolism and apoptosis. The crucial role in cell fate determination has long made VDAC1 a promising target in cancer research. The recent discovery of a highly conserved and druggable NADH‐like binding pocket has led to the development of specific VDAC antagonists (VA) with potential antitumor activity. Here, we performed electrophysiological analysis in artificial lipid membranes to examine in detail how these drugs affect VDAC1 gating. Upon addition of VA molecules to a planar bilayer containing recombinant human VDAC1, single channel recordings showed a reliable reduction in the voltage dependence of the pore. Experiments performed in asymmetric KCl solution revealed that VA binding renders the channel predominantly anion selective, potentially disrupting cation fluxes and simultaneously affecting the transport of negatively charged metabolites. Taken together, these data represent a step forward into the comprehension of VDAC modulation as a potential therapeutic approach in cancer management.

## Graphical abstract

VA molecules alter VDAC1 gating by increasing anion flow and reducing cation permeability. In cancer cells, which rely on ER‐mitochondria Ca^2+^ transfer and overexpress VDAC1, this imbalance triggers bioenergetic stress, ROS buildup, and mitochondrial collapse, leading to cell death. In normal cells – where VDAC1 is not overexpressed – changes in channel ion conductance do not impair mitochondrial function. Rather, they slow proliferation and halt cell cycle progression, preventing energetic failure.

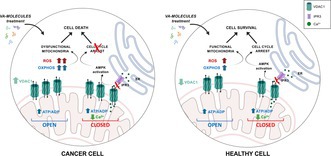

**Abbreviations**
ADP: Adenosine Diphosphate
AMPK: AMP‐Activated Protein Kinase
ATP: Adenosine Triphosphate
CK: Creatine Kinase
EC50: Half Maximal Effective Concentration (Effective Concentration 50)
EDTA: Ethylenediaminetetraacetic acid
ER: Endoplasmic Reticulum
G: Conductance
G_0_
: Maximal Conductance
Grp75: Glucose‐Regulated Protein 75
HK: Hexokinase
IP3R: Inositol 1,4,5‐trisphosphate Receptor
LDAO: Lauryldimethylamine‐N‐oxide
MERCs: Mitochondria‐ER Contact Sites
mTOR: Mammalian Target of Rapamycin
NaCl: Sodium Chloride
NADH: Nicotinamide Adenine Dinucleotide
OMM: Outer Mitochondrial Membrane
PLB: Planar Lipid Bilayer
P_open_ (P_o_): Open Probability
ROS: Reactive Oxygen Species
SODI: Superoxide Dismutase I
TRIS/HCl: Tris(hydroxymethyl)aminomethane hydrochloride
VA: VDAC Antagonist
VDAC: Voltage‐Dependent Anion Selective Channel
V_dep_
: Voltage Dependence
Δ*ψ*_m_
: Mitochondrial Membrane Potential

## Introduction

VDACs are abundant OMM proteins in charge of controlling ions, respiratory substrates, ATP, ADP, and inorganic phosphate exchanges across the organelle [1, 2]. In mammals, three evolutionary conserved VDACs (i.e., *VDAC1*, 2, and 3) share approximately 70% sequence similarity and accomplish distinct cellular tasks [3, 4, 5, 6]. Among them, VDAC1 is undoubtedly the most prevalent and extensively characterized isoform [7, 8, 9, 10, 11, 12, 13]. At low or zero membrane potentials, VDAC1 channels reconstituted into artificial lipid bilayer adopt a high‐conductance “open” state that has been fully resolved [14, 15, 16]. Positive and negative voltages higher than 30–40 mV symmetrically switch VDAC1 to multiple structurally uncharacterized low‐conductance states [7, 17], both decreasing pore diameter and permeability to large anionic metabolites, primarily ATP. The so‐called “closed” states slightly prefer cations, but still allow the passage of small anions such as Cl^−^. To date, the mechanism underlying the gating of VDAC1 is not yet completely deciphered, albeit most of the literature data agree that the N‐terminal α‐helix plays a key role in this process [9, 15, 16, 18, 19]. VDAC1 is implicated in a broad spectrum of cellular pathways, including apoptosis [20], ferroptosis [21, 22, 23], calcium homeostasis, and ROS signaling [6]. These functions rely on its capacity to interact with key cytosolic enzymes (such as hexokinases I/II and creatine kinase) as well as with both pro‐ and anti‐apoptotic factors (i.e., Bax, Bcl‐2, and Bcl‐xL). In light of its multiple roles, VDAC1 has long been considered a critical pharmacological target in various diseases, such as cancer and neurodegenerative disorders [24, 25]. Notwithstanding, the development of VDAC interacting compounds has been significantly hampered by the lack of well‐defined druggable binding sites and the existence of isoform specificity. Most currently available molecules and peptides targeting VDAC1 act non‐specifically: they modulate channel activity [26, 27, 28, 29], antagonize the interaction with hexokinases (HKI‐II) [30, 31, 32] and superoxide dismutase I (SODI) [10] or even regulate protein expression [33, 34, 35]. Lately, the identification of a NADH‐binding pocket on the inner wall of VDAC1, highly conserved among the three isoforms, paved the way for the design of *ad hoc* compounds that have proven to selectively induce cancer cell death [36, 37, 38, 39, 40]. Physiologically, NADH promotes channel closure by steric hindrance and electrostatic repulsion [36, 41], contributing to partially suppress mitochondrial metabolism [42]. In tumors, this condition fuels the Warburg phenotype wherein cells rapidly ferment glucose to lactic acid rather than oxidize it via mitochondria even in the presence of oxygen. Hence, in this *scenario*, molecules that interfere/compete with the binding of NADH have been proposed to stabilize the VDAC1 pore in the open state, forcing cancer cells to switch energy production towards oxidative phosphorylation [36, 37, 38, 39, 40]. Previously, we identified five small molecules with a three‐ringed architecture *in silico*‐designed to fit within the NADH‐binding pocket of VDAC1 (i.e., VDAC antagonist (VA) molecules) that exhibited remarkable antiproliferative activity both in cancer cell lines and in living organoids [40]. However, how these drugs affect *VDAC* ion channel properties and modulate its permeability have remained unexplored. To address this, we here provide a detailed biophysical characterization of VA molecules and human VDAC1 (hVDAC1) interaction using the planar lipid bilayer technique. Single‐channel recordings demonstrated that all five tested drugs interfered with the voltage gating of the channel reconstituted into an artificial membrane and concurrently helped to stabilize the “open” state. Strikingly, VA molecules also induced a thorough loss of cation preference in the closed states that turned hVDAC1 into an almost non‐selective channel. Overall, data collected contribute to the understanding of the metabolic changes prompted by VDAC‐targeted molecules and lay the basis for their structure‐aided chemical optimization aimed at developing future novel specific cancer therapeutics.

## Results

### β‐NADH reduces hVDAC1 conductance at low membrane potentials

**Fig. 1:**
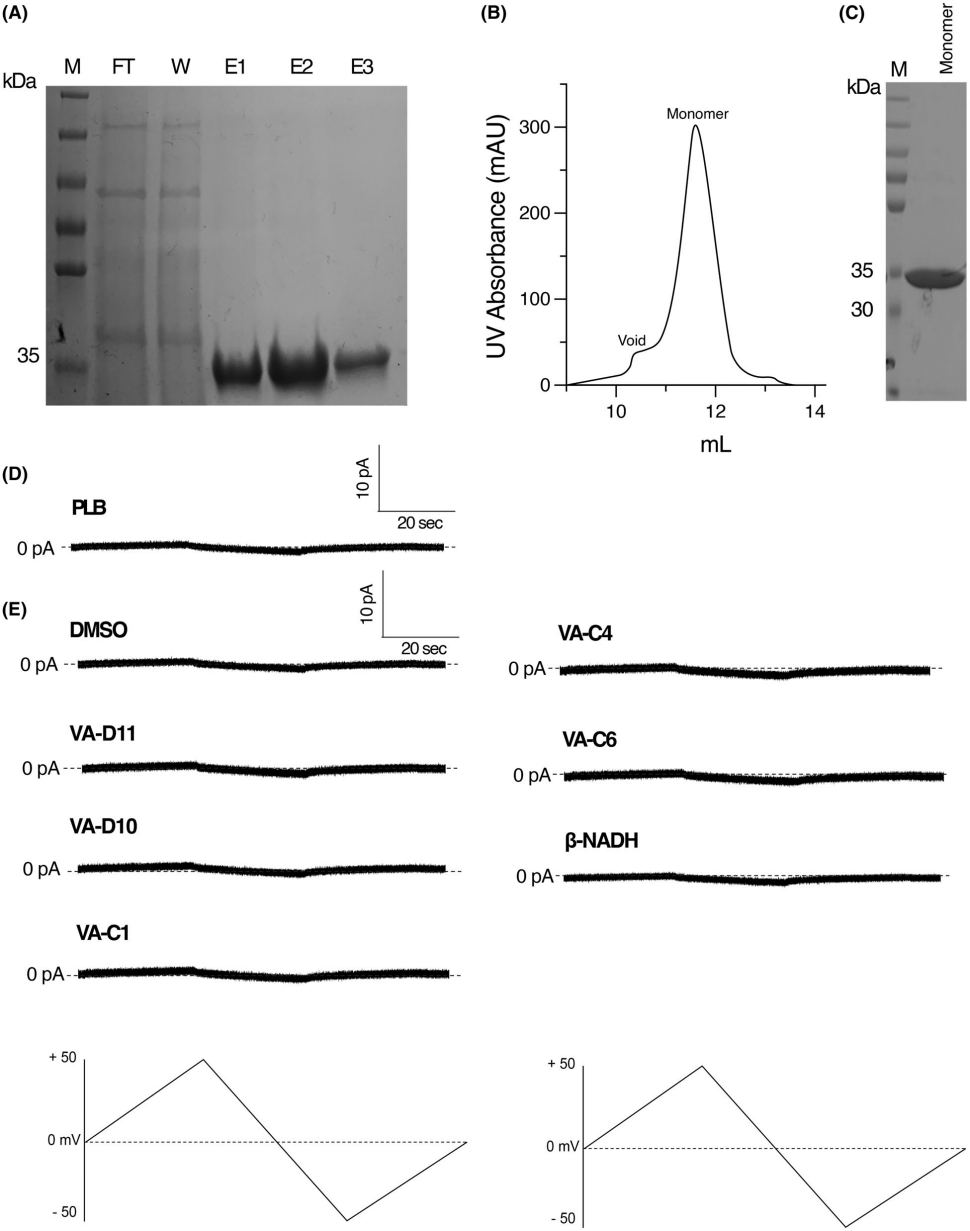
Purification of recombinant His‐tagged human VDAC1 and analysis of the effects of individual compounds on planar lipid membrane stability. (A) SDS/PAGE analysis of Ni‐NTA affinity chromatography fractions. Molecular weight marker (M); flow through (F); wash (W); elution fractions (E1–E3). (B) Size‐exclusion chromatography (SEC) profile of refolded hVDAC1. The ‘void’ peak corresponds to protein aggregates, the ‘monomer’ peak represents monomeric hVDAC1. (C) SDS/PAGE of pooled monomeric SEC fractions (Monomer); molecular weight marker (M). (D) Control recording of an asolectin bilayer membrane without VA (VDAC Antagonist) molecules, β‐NADH, or DMSO. (E) Electrophysiological recordings after addition of different compounds to both sides of the bilayer: VA molecules (20 μm), β‐NADH (15 μm), or DMSO (0.6%). Current stability was measured using a ± 50 mV voltage ramp in symmetrical buffer (1 m KCl, 1 mm Hepes, pH 7.4) and compared to the control (D).

**Fig. 2:**
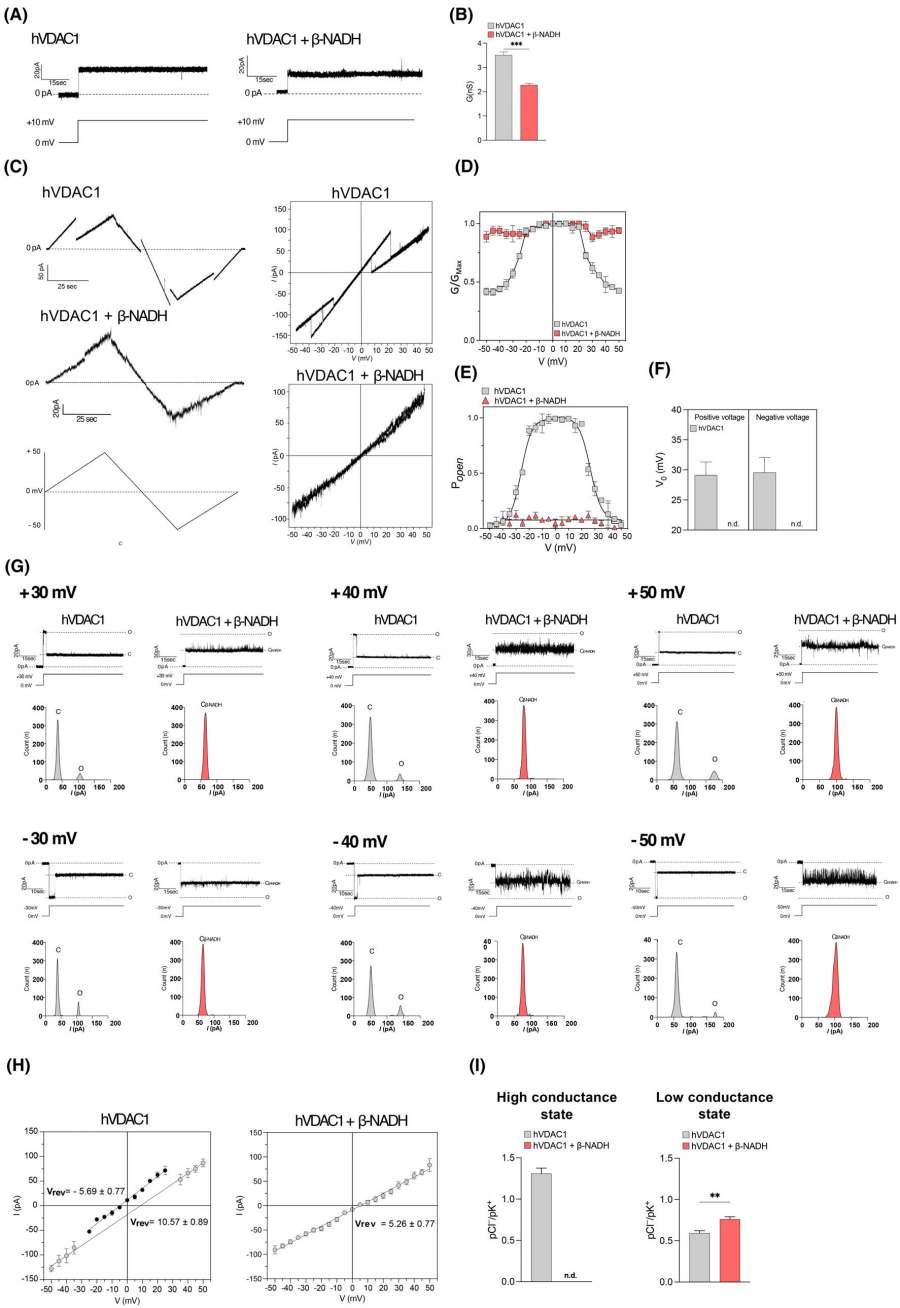
Electrophysiological properties of recombinant human VDAC1 in the presence/absence of β‐NADH. (A) Representative current traces of single hVDAC1 channels recorded at +10 mV in symmetrical 1 m KCl before and after addition of 15 μm β‐NADH to both sides of the bilayer. (B) Representative histograms of hVDAC1 conductance at +10 mV in the presence or absence of 15 μm β‐NADH. Data are represented as mean ± SD of *n* = 6 independent experiments. Statistical analysis was performed using an unpaired *t*‐test; ***P < 0.001. (C) Single‐channel current traces of hVDAC1 in symmetrical 1 m KCl upon application of a triangular voltage ramp before and after 15 μm β‐NADH addition. Corresponding I–V plots show current as a function of applied voltage. (D) Relative conductance (G/G_max_) of apo and NADH‐treated hVDAC1 channels as a function of applied voltage. (E) Open probability (P_open_) as a function of the applied voltage before and after treatment. (F) Histograms show the gating voltage (V₀) corresponding to the open‐to‐closed transition. Data are represented as mean ± SD of *n* = 6 independent experiments. Statistical analysis was conducted using one‐way ANOVA followed by Tukey's test. (G) Representative current traces of single hVDAC1 channels before and after addition of 15 μm β‐NADH, recorded at ±30, ±40, and ± 50 mV in symmetrical 1 m KCl. Quantitative analysis of conductance at each voltage is shown: gray, apo‐hVDAC1; pink, β‐NADH–treated (15 μm). Data are represented as mean ± SD of *n* = 6 independent experiments. (H) I–V plots of hVDAC1 recorded in a 10‐fold KCl gradient (0.1/1 m) before and after β‐NADH addition. Solid lines indicate regression fits for the high‐conductance (O, black) and low‐conductance (C, gray) states of apo‐hVDAC1, and the single state observed in the presence of β‐NADH (pink). Data are represented as mean ± SD of *n* = 6 independent experiments. (I) Permeability ratios (P_Cl_^−^/P_K_^+^) calculated from reversal potentials using the Goldman–Hodgkin–Katz equation before and after β‐NADH treatment. Data are represented as mean ± SD of *n* = 6 independent experiments. Statistical analysis was performed using an unpaired *t*‐test; *n.d*, not determined; **P < 0.01.

VA molecules and β‐NADH, a VDAC1 naturally occurring modulator, share part of the same binding pocket [40]. As a result, the effect of β‐NADH on VDAC's gating was first evaluated using a planar lipid bilayer (PLB) assay [43], providing a baseline for subsequent measurements of the VA molecules. Accordingly, recombinant human VDAC1 was expressed in *E. coli*, purified by Ni‐NTA affinity chromatography (Fig. 1A), and the monomeric fraction obtained by size‐exclusion chromatography was incorporated into PLB membrane (Fig. 1B,C). As shown in Fig. 1D,E, 15 μM β‐NADH, a concentration previously established by Zizi *et al*. [36], did not alter the artificial membrane alone as no background currents were detected over the recording time. Though, it produced a significant reduction in channel conductance (from 3.45 ± 0.16 nS to 2.23 ± 0.11 nS) when added to both sides of a single hVDAC1 pore embedded into a lipid bilayer in symmetric 1 m KCl with an input voltage of +10 mV (Fig. 2A,B). Occupancy of the binding pocket by β‐NADH also prevented the channel from undergoing closure at membrane voltages that normally induce pore changes toward the low‐conductance states (Fig. 2C–F), concurrently increasing the current noise especially at higher voltages, in agreement with [44]. Consistent with this observation, hVDAC1 maintained a long‐standing cation selectivity across all tested voltages without ever reaching any preference for the passage of anions (Fig. 2G–I).

### VA molecules do not affect the high‐conductance open state of the channel

**Fig. 3:**
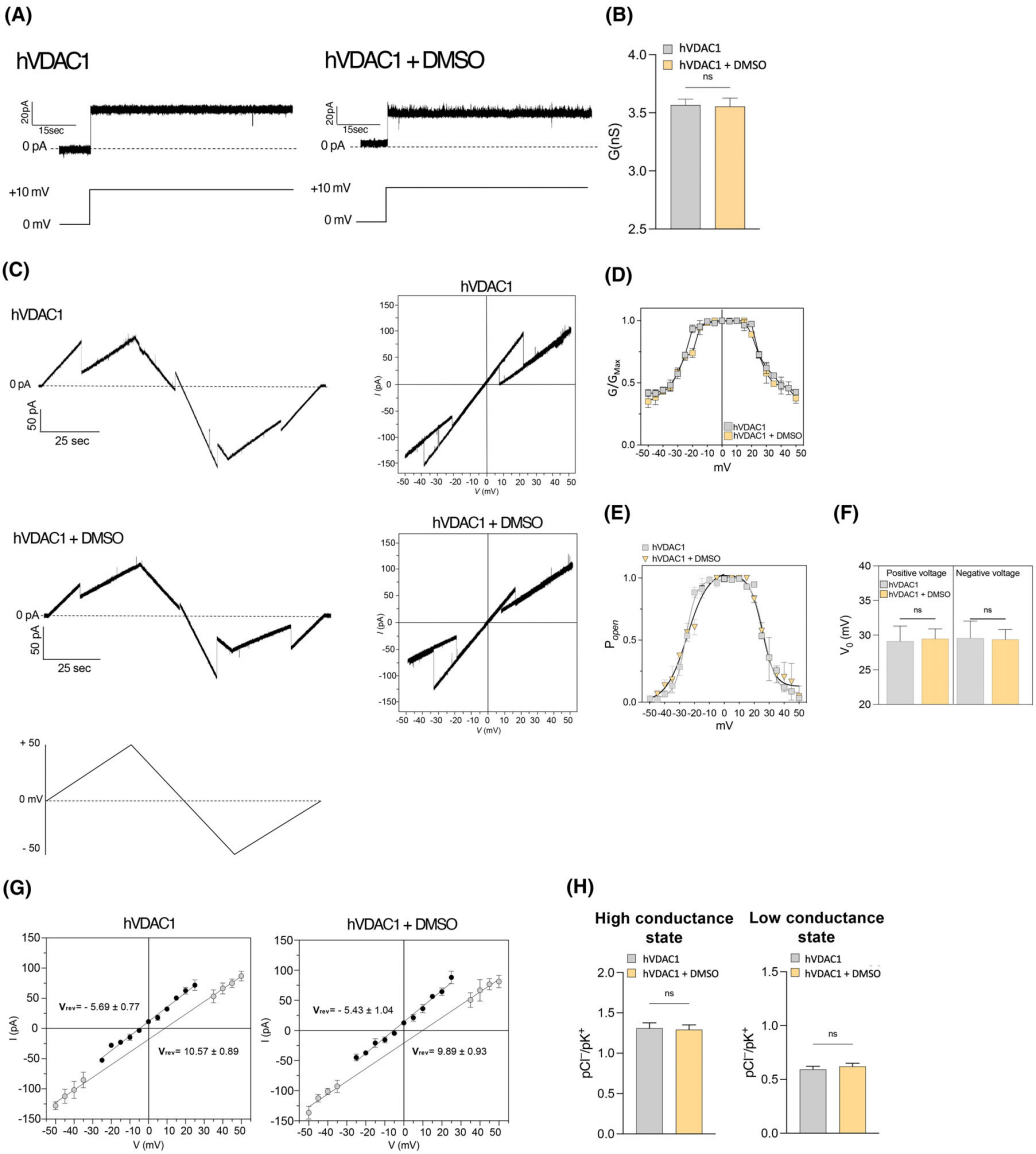
Electrophysiological properties of recombinant human VDAC1 in the presence/absence of DMSO. (A) Representative single‐channel current traces of hVDAC1 at +10 mV in symmetrical 1 m KCl before and after the addition of 0.6% DMSO. (B) Representative histograms of hVDAC1 conductance at +10 mV in the presence or absence of DMSO. Data are represented as mean ± SD of *n* = 4 independent experiments. Statistical analysis was performed using unpaired *t*‐test; *ns*, not significant. (C) Single‐channel recordings of hVDAC1 upon application of a triangular voltage ramp in symmetrical 1 m KCl before and after addition of 0.6% DMSO. Corresponding I–V plots show current as a function of applied voltage. (D) Relative conductance (G/G_max_) as a function of applied voltage for the apo and DMSO‐treated channels. (E) Open probability (P_open_) as a function of the applied voltage before and after treatment. (F) Histograms show the gating voltage (V₀) corresponding to the open‐to‐closed transition. Data are represented as mean ± SD of *n* = 3 independent experiments. Statistical analysis was conducted using one‐way ANOVA followed by Tukey test. (G) I–V plots of hVDAC1 recorded in a 10‐fold KCl gradient (0.1/1 m) before and after DMSO addition. Solid lines represent regression fits for the high‐conductance (O, black) and low‐conductance (C, gray) states. Data are represented as mean ± SD of *n* = 3 independent experiments. (H) Permeability ratios (P_Cl_^−^/P_K_^+^) calculated from reversal potentials using the Goldman–Hodgkin–Katz equation before and after 0.6% DMSO treatment. Statistical analysis was performed using unpaired *t*‐test; *ns*, not significant.

**Fig. 4:**
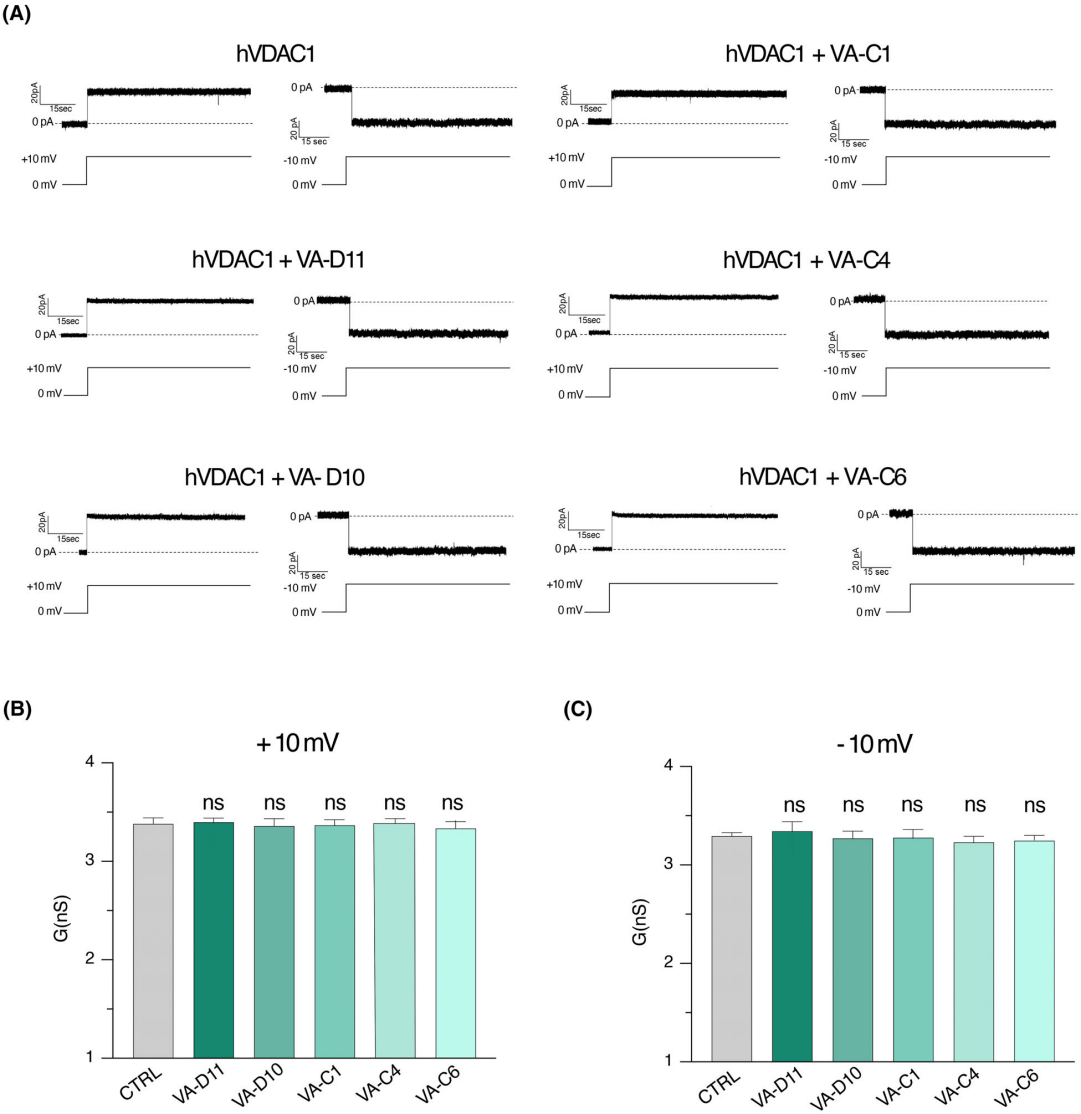
Current traces of human VDAC1 single channel in the presence of VA (VDAC Antagonist) molecules. (A) Representative current traces of single hVDAC1 channels recorded at ±10 mV in symmetrical 1 m KCl before and after addition of 20 μm VA molecules. (B, C) Representative conductance histograms of hVDAC1 at ±10 mV in the presence or absence of VA molecules. Data are represented as mean ± SD of *n* = 4 independent experiments. Statistical analysis was conducted using one‐way ANOVA followed by Tukey's test; *ns*, not significant.

The stability of the phospholipid bilayer was first monitored upon addition of the VA molecules and the vehicle solvent (DMSO) alone to both *cis* and *trans* sides of the cuvette at the final concentration of 20 μm and 0.6%, respectively, in order to exclude interference‐type noise. As displayed in Fig. 1, no deviations from the baseline current (0 pA) were recorded upon application of a triangular voltage wave of ±50 mV in the presence of DMSO (Fig. 1D,E) or the tested molecules (Fig. 1D,E), suggesting that the membrane integrity was unaffected throughout the entire analysis. Analogously, DMSO did not perturb the biophysical properties of membrane‐embedded hVDAC1 that rather maintained unchanged conductance, voltage dependence and ion selectivity (Fig. 3A–H), therefore allowing further PLB analysis of pore activity to be carried out with the VA molecules. Accordingly, channel conductance (G) was investigated before and after the addition of 20 μm of each of the five VDAC‐targeted drugs to both sides of a planar bilayer containing a single hVDAC1 pore in symmetric 1 m KCl with a constant applied voltage of ±10 mV. 20 μm was specifically selected as a saturating concentration based on the EC50 determination of each molecule at the planar lipid bilayer (not shown) and the K_D_ values measured in [40]. As shown in the first current trace of Fig. 4A, in the absence of the molecules hVDAC1 stably persisted in a high‐conductance open state with an average value of G = 3.45 ± 0.16 nS, in accordance with results reported elsewhere [7, 10, 13, 45]. The current amplitude of the open state was not significantly altered by any of the VA molecules examined (Fig. 4A), as clearly illustrated in Fig. 4B,C by the average conductance values of hVDAC1 calculated following the addition of each compound and plotted against the corresponding G value of the untreated channel (VA‐D11 = 3.41 ± 0.17; VA‐D10 = 3.42 ± 0.12; VA‐C1 = 3.44 ± 0.14; VA‐C4 = 3.48 ± 0.12; VA‐C6 = 3.40 ± 0.36). These data indicate that molecules targeting the NADH‐binding site of VDAC1 do not affect the maximal conductance of the channel at low membrane potentials.

### VA molecules impair voltage response of human VDAC1

**Fig. 5:**
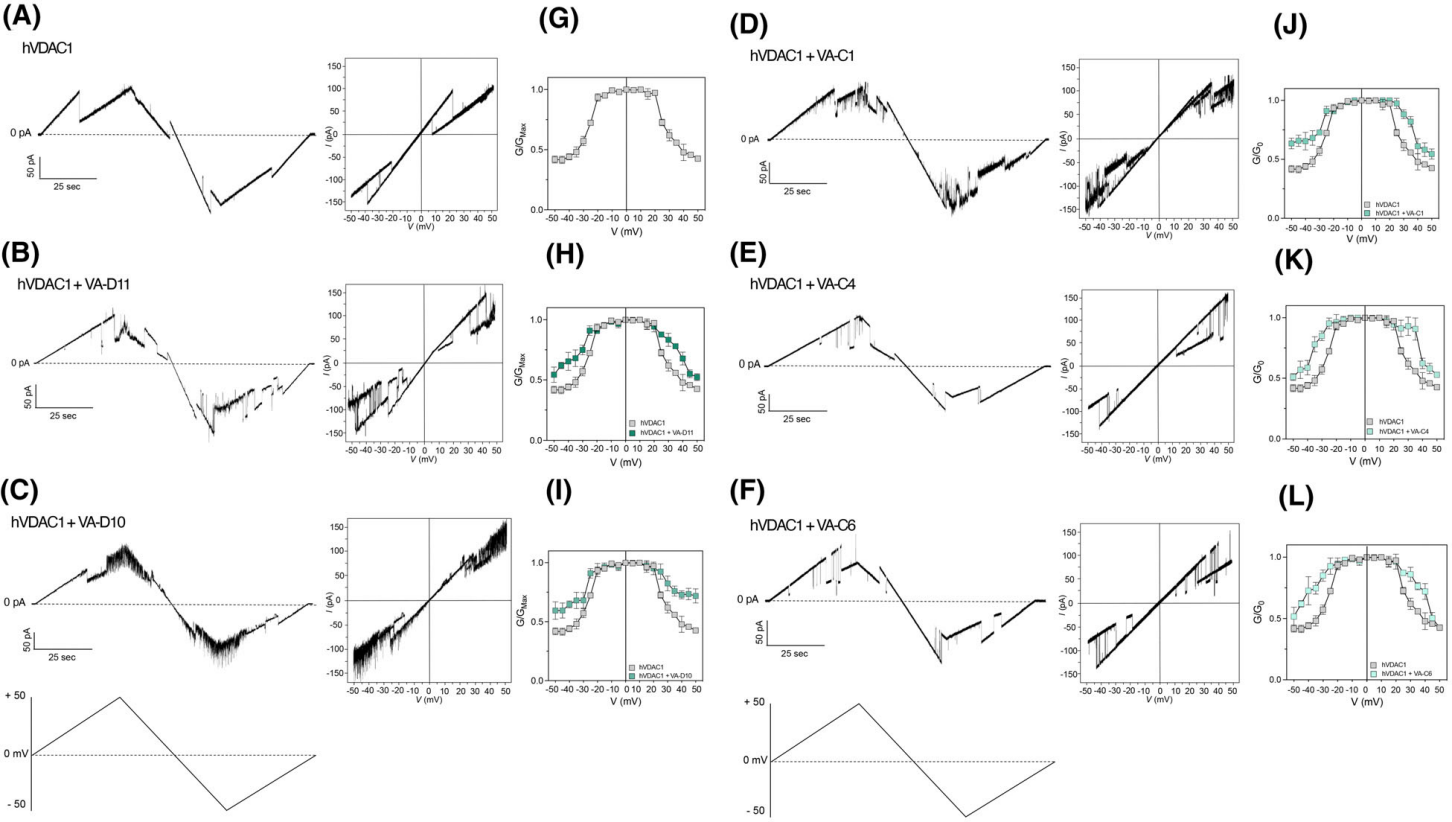
Effects of VA (VDAC Antagonist) molecules on hVDAC1 voltage dependence. Single‐channel current traces of hVDAC1 upon application of a triangular voltage ramp in symmetrical 1 m KCl. Corresponding I–V plots show current as a function of applied voltage. (A) apo‐hVDAC1; (B) hVDAC1 + VA‐D11; (C) hVDAC1 + VA‐D10; (D) hVDAC1 + VA‐C1; (E) hVDAC1 + VA‐C4; (F) hVDAC1 + VA‐C6. Relative conductance (G/G_max_) as a function of applied voltage for the apo (G) and treated hVDAC1 (H–L). Data are represented as mean ± SD of *n* = 4 independent experiments.

The permeability of VDAC to ions and metabolites dynamically responds to changes in membrane potential [17, 46]. At low or zero membrane potentials, the pore conductance raises linearly with the voltage and perdures in the high‐conductance open state fully described in the previous paragraph. The application of potentials higher than ±30 mV induces a step‐like transition towards multiple closed states with a large drop in conductance [10, 13, 19]. To investigate the impact of the VA molecules on the voltage gating of the channel, a triangular voltage wave with an amplitude of ±50 mV was applied to human recombinant VDAC1 reconstituted in artificial membrane before and after the addition of the tested compounds (Fig. 5), and the derived current/voltage (I/V) plots were analyzed. In these experimental conditions, untreated hVDAC1 stably transitioned from the open to the closed states at ±20–30 mV (Fig. 5A). Plots of the normalized average VDAC conductance (G/G_max_) as a function of the applied voltage demonstrated the apo form of the channel exhibited the typical bell‐shaped curve (i.e., lower conductance values at higher membrane potentials) that reflects the symmetry of the voltage‐gating process (Fig. 5G). The addition of each of the VDAC antagonists shifted the channel closure to potentials higher than ±30 mV and induced fast flickering/transition between substates in the channel activity both at positive and negative applied voltages (Fig. 5B–F). In this context, all the five molecules reduced the steepness of the bell‐shaped curve, although D11 and C1 provoked the greater loss of voltage response at negative or positive applied voltages, respectively (Fig. 5H–L). The percentage decrease in hVDAC1 voltage dependence upon treatment with the VA molecules ranged from approx. 25% (VA‐D11) to 30–32% (VA‐D10, VA‐C1, VA‐C4, VA‐C6), suggesting they directly interfere with the gating mechanism of pore by weakening the protein sensitivity to the membrane potential as shown in Table 1. In contrast, β‐NADH maintained the channel in a persistently low‐conductance state, exhibiting no variation in the voltage‐dependent gating, as shown in Fig. 2C,D.

**Table 1:** Percentage of reduction of hVDAC1 voltage dependence (V_dep_) upon addition of the VA (VDAC Antagonist) drugs. Quantification of hVDAC1 voltage dependence reduction after the treatment with the different compounds tested. The reduction of V_dep_ is calculated from the G/G_max_ curves compared to the untreated protein. Data are represented as mean ± SD of *n* = 3 independent experiments.

Reduction of VDAC1 V_dep_ (Vm)				
VA‐D11	VA‐D10	VA‐C1	VA‐C4	VA‐C6
25.48 ± 3.04%	30.90 ± 2.50%	30.28 ± 2.43%	32.96 ± 2.97%	32.73 ± 2.51%

### VA molecules force hVDAC1 in the open state even at high voltages

**Fig. 6:**
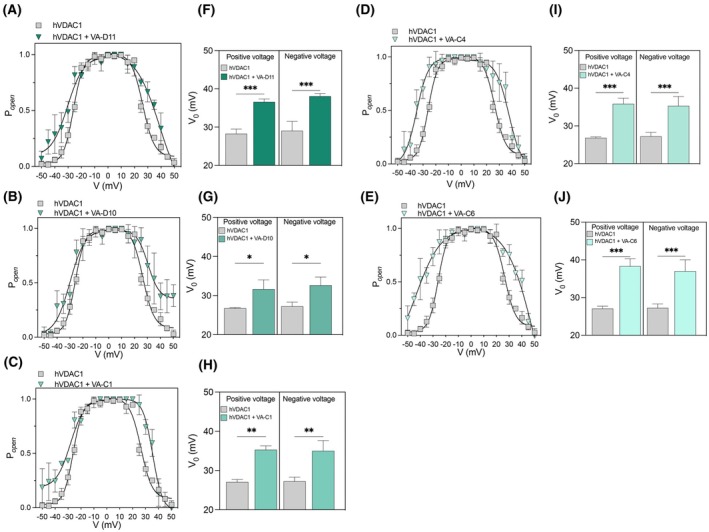
Effects of VA (VDAC Antagonist) molecules on hVDAC1 open probability. Open probability (P_open_) as a function of applied voltage before and after treatment with VA molecules (A–E). Histograms show the gating voltage (V₀) corresponding to the open‐to‐closed transition (F–J). Data are represented as mean ± SD of *n* = 3 independent experiments. Statistical analysis was conducted using one‐way ANOVA followed by Tukey's test (*P < 0.05; **P < 0.01; ***P < 0.001).

**Fig. 7:**
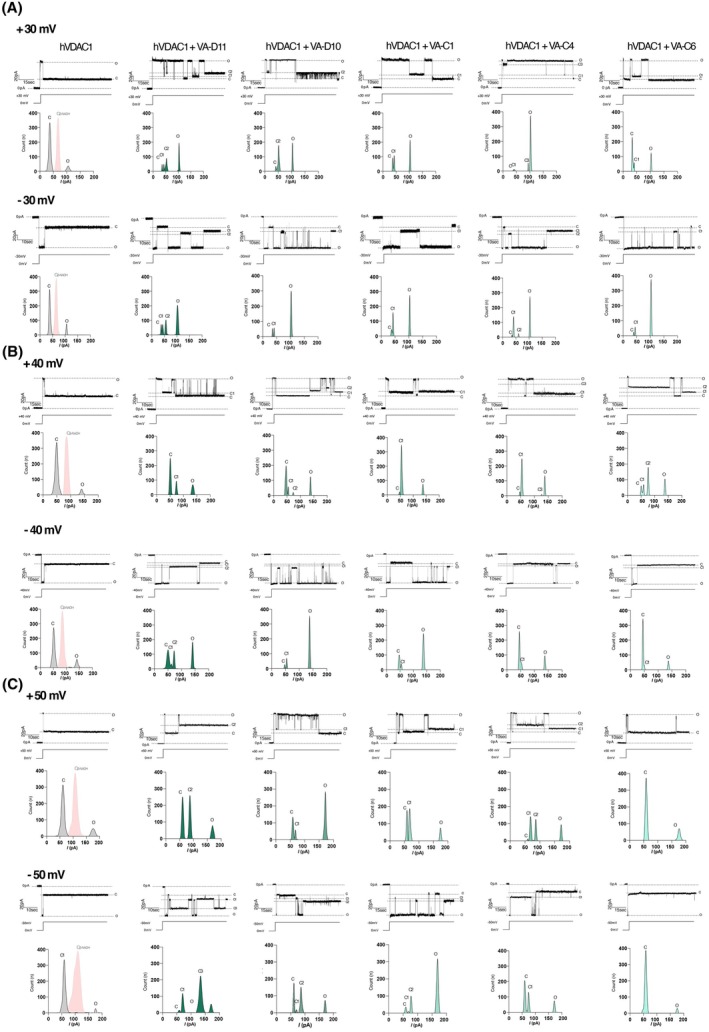
Effects of VA (VDAC Antagonist) molecules on hVDAC1 voltage gating behavior. (A–C) Representative current traces of single hVDAC1 channels before and after addition of 20 μM VA molecules, recorded at constant voltages (±30, ±40, and ± 50 mV) in symmetrical 1 M KCl. High conductance state (O); low conductance state (C); intermediate low‐conductance states (C1 and C2); β‐NADH‐induced low conductance state (C_β‐NADH_). Quantitative analysis of conductance events at each voltage is shown: gray, apo‐hVDAC1; black, VA‐treated. Data are represented as mean ± SD of *n* = 3 independent experiments.

**Fig. 8:**
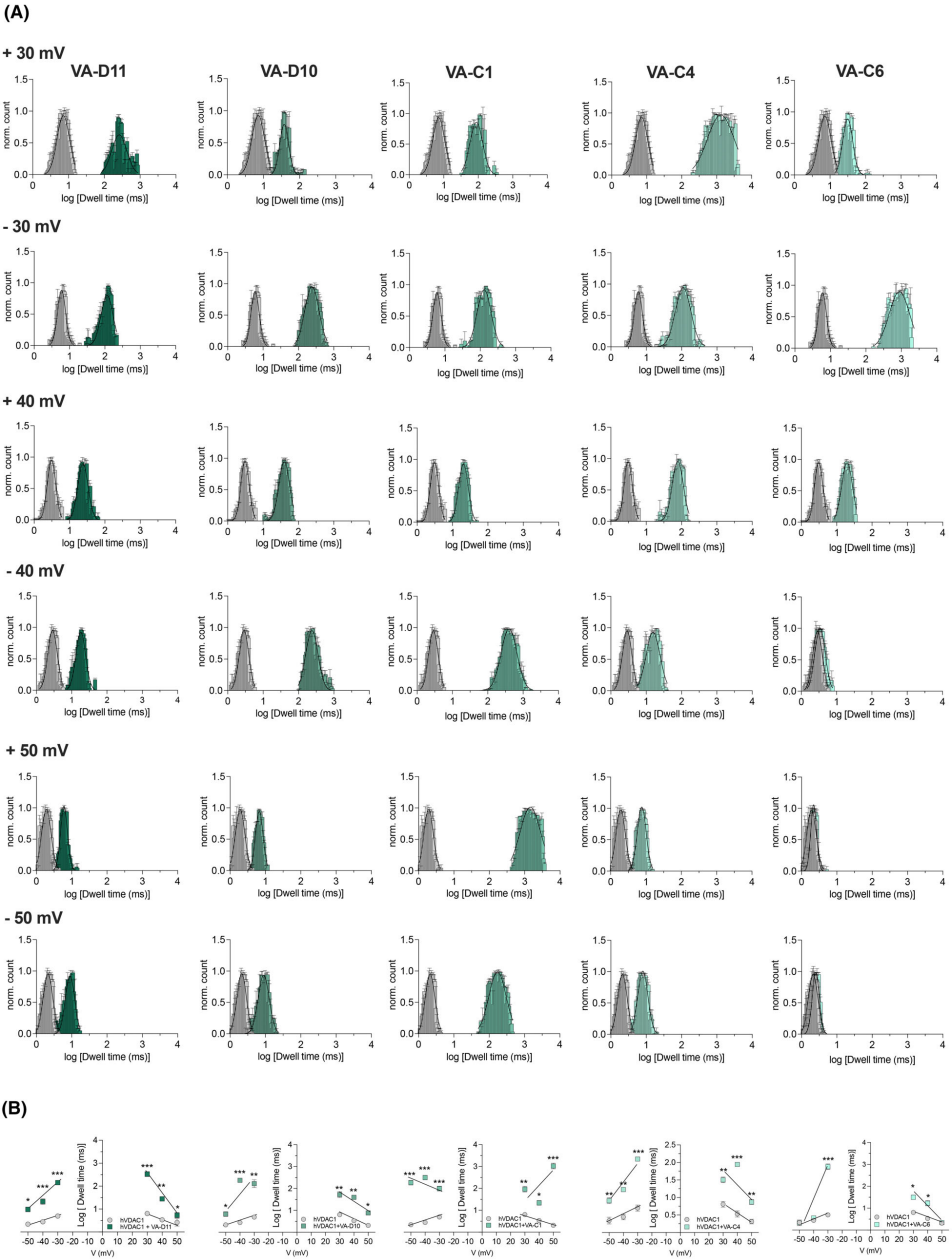
Dwell time analysis of hVDAC1 single channel in the presence of VA (VDAC Antagonist) molecules. (A) Histogram of dwell times for hVDAC1 apo (gray) and treated with VA molecules (green gradient) at various constant voltages. Lines show log‐normal fits; distribution peaks indicate the most probable dwell times. (B) Dwell time versus applied voltage. *Solid lines* represent linear regressions for apo‐hVDAC1 (gray) and treated hVDAC1 (green gradient). Data are represented as mean ± SD of *n* = 3 independent experiments. Statistical analysis was conducted using unpaired *t*‐test; *P < 0.05, **P < 0.01, ***P < 0.001.

The open probability (P_open_) was calculated before and after VA molecules administration to either side of the membrane containing a single hVDAC1 channel (Fig. 6A–E). Again, each of the drugs led to less steep bell‐shaped curves compared to untreated hVDAC1. The voltage values associated with the transition of hVDAC1 from the open to the closed state (V_0_) were indeed significantly higher in the presence of the VA molecules (Fig. 6F–J). Notably, VA‐D11 and VA‐D10 raised the recorded V_0_ to values greater than ±30 mV, while VA‐C1, VA‐C4, and VA‐C6 increased it to values over ±35 mV. The constant low‐conductance state of hVDAC1 registered in the presence of β‐NADH prevents us from calculating the open probability (Fig. 2E,F). Considering the results obtained, we thoroughly investigated the behavior of the mitochondrial porin at membrane potentials that normally switch the channel to stable low conductance states, by applying voltage steps of ±10 mV over a range of ±30–50 mV for 40 s, as shown by the representative current traces depicted in Fig. 7A–C. At high potentials applied (≥ ± 30 mV), apo‐hVDAC1 underwent fast gating from high‐ (O) to low‐conductance (C) state, reaching an average value of G = 1.29 ± 0.45 nS. β‐NADH binding reduced the channel conductance to 2.13 ± 0.56 nS (C_β‐NADH_) (Fig. 2F), whereas each of the small molecules shifted VDAC1 to different sub‐closed states (C1, C2, and C3) characterized by higher conductance values than C. Histograms of the G values as a function of the number of events clearly illustrate how many times each analyzed state occurs over the recording period (Fig. 7A–C): as can be appreciated, the unique peak corresponding to the closed state of untreated hVDAC1 (C), alongside changing its frequency distribution, is flanked by C1 or C2 and C3 additional peaks upon the addition of VA‐D11, VA‐D10, VA‐C1, VA‐C4, VA‐C6 or VA‐D11, VA‐C4, respectively. At the same time, the VA molecules increase the number of counts corresponding to the open state (O) of apo‐hVDAC1 at voltages that should properly reduce it. However, whereas VA‐D11, VA‐D10, VA‐C1, and VA‐C4 altered mitochondrial porin kinetics even at membrane potentials of ±50 mV, VA‐C6 maintained unchanged the voltage response of hVDAC1 at voltages above ±40 mV (Fig. 5C). The tendency of the molecules to promote hVDAC1 in the open state and to destabilize the channel under high applied voltages is further supported by the analysis of the distribution of open‐state dwell times. The log‐binned distributions of hVDAC1 open times obtained with 20 μm of the VA molecules across different ranges of applied potentials – derived from statistical analysis of the corresponding current traces – reveal a rightward shift in the distribution, indicative of prolonged channel opening (Fig. 8A). Notably, all tested molecules elicited a comparable effect, producing a shift of approximately 1–3 log units, although VA‐C6 did not induce significant changes at membrane potentials of −40 and ± 50 mV. Linear regression analysis of hVDAC1 open‐state dwell times at both positive and negative potentials demonstrates to an even greater extent the modulatory effects of the tested molecules on the channel (Fig. 8B). In detail, the apo form of hVDAC1 exhibited a modest negative slope (β = −0.0195 ± 0.0048, p = 0.0046) at positive potentials, whereas at negative potentials it displayed a positive slope (β = 0.0178 ± 0.0037). In the presence of the VA drugs, the regression lines were shifted upward, indicating enhanced stability of the open state. In particular, VA‐D11, VA‐D10, and VA‐C4 induced a slope increase of approximately 0.01–0.04 units relative to the untreated control. VA‐C1 led to a variation in the slope at both positive and negative potentials (β = 0.0530 ± 0.0256 and β = −0.0133 ± 0.0090, respectively), suggesting that this molecule has a greater ability to maintain the channel in the open state, even at high voltages. Conversely, although VA‐C6 altered the slope, the dwell time values were not significantly different from those of apo‐hVDAC1 at −40 and ± 50 mV, confirming the findings shown in Fig. 8A.

### VA molecules partially suppress the cation selectivity of recombinant human VDAC1

**Fig. 9:**
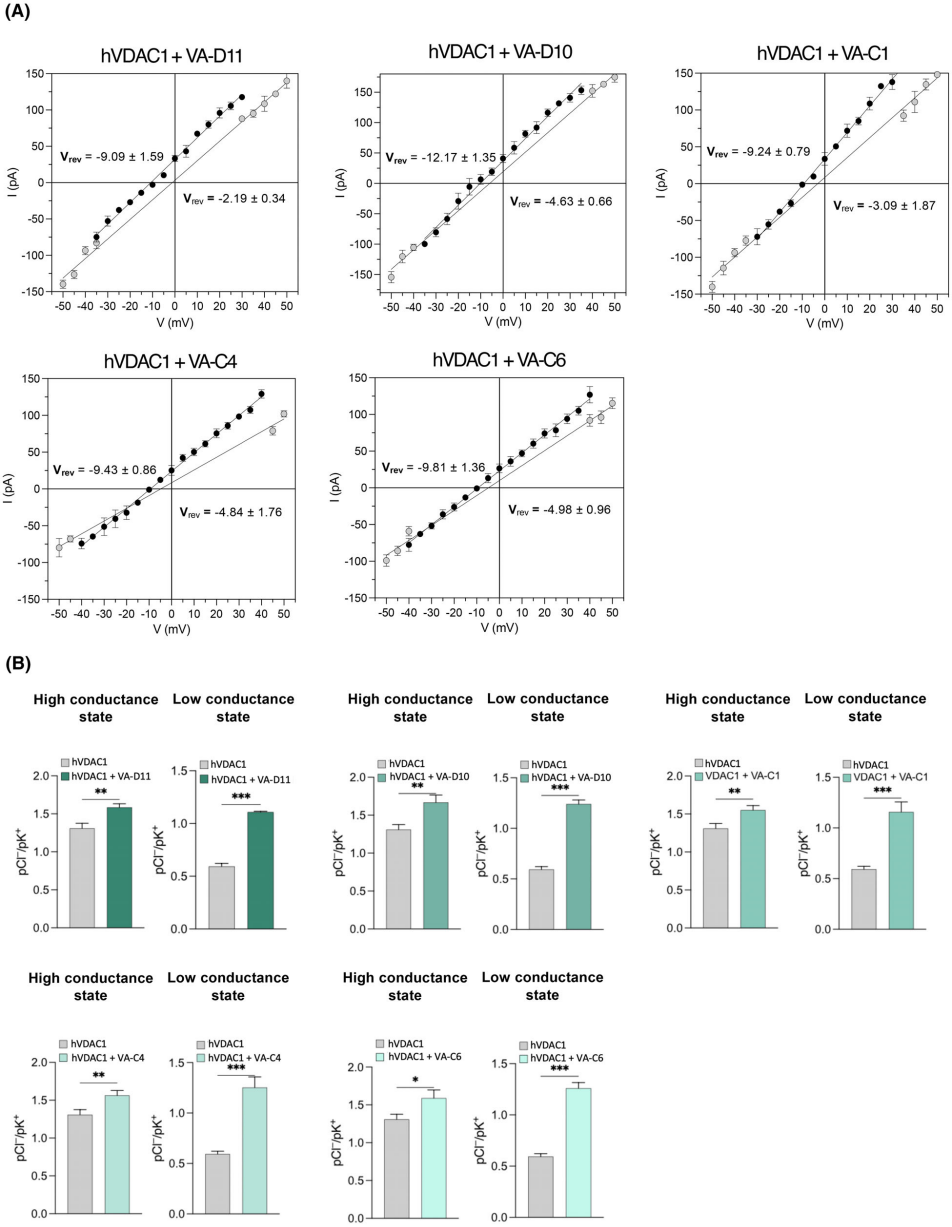
Ion selectivity analysis of hVDAC1 single channel in the presence of VA (VDAC Antagonist) molecules. (A) I/V curves of recombinant human VDAC1 recorded in a 10‐fold KCl gradient (0.1/1 m) before and after addition of VA molecules. *Solid lines* represent regression fits for the high‐conductance (O, black) and low‐conductance (C, gray) states. Data are represented as mean ± SD of *n* = 3 independent experiments. (B) Permeability ratios (P_Cl_^−^/P_K_^+^) calculated from reversal potentials using the Goldman–Hodgkin–Katz equation for human VDAC1 in high‐ and low‐conductance states before and after addition of 20 μm VA molecules. Data are represented as mean ± SD of *n* = 3 independent experiments. Statistical analysis was performed using unpaired *t*‐test; *P < 0.05, **P < 0.01, ***P < 0.001.

VDAC1 exhibits a slight preference for anions over cations in the “open state”, whereas the “closed states” favor non‐selective permeation of cations including Na^+^, K^+^, and Ca^2+^ [47, 48]. Herein, the ion selectivity of human recombinant VDAC1 was explored at the planar lipid bilayer in asymmetric KCl concentrations (1 m *cis* /0.1 m *trans*) following application of a triangular voltage wave of ±50 mV. The asymmetric buffer condition allows to calculate the reversal potential (V_r_), consisting in the voltage value that corresponds to zero current used to calculate the permeability ratio of Cl^−^ to K^+^ (P_Cl_^−^/P_K_^+^) in both high‐ and low‐conductance states by the Goldman‐Hodgkin‐Katz equation. Under these experimental conditions, apo‐hVDAC1 displayed the typical anion selectivity in the open state with a negative V_r_ = −5.69 ± 0.77 mV and a slight preference towards cations in the closed states characterized by a positive V_r_ = 10.57 ± 0.89 mV, corresponding to a permeability ratio of P_Cl_^−^/P_K_^+^ = 1.31 ± 0.06 and P_Cl_^−^/P_K_^+^ = 0.59 ± 0.03, respectively (Fig. 9A,B). The addition of the VA molecules to reconstituted hVDAC1 led to an extreme upheaval of the channel selectivity characterized by an increase of the anion selectivity at low membrane potentials ranging from 24% to 36% and a 55% to 67% loss of the cation preference at high membrane potentials (Fig. 9A,B). The permeability ratios of the open and closed states before and after the administration of the VA molecules are reported in Table 2.

**Table 2:** Permeability ratios of the open and closed states of hVDAC1 in the presence or absence of the VA (VDAC Antagonist) molecules. The permeability ratios (P_Cl+_ /P_K−_) of recombinant hVDAC1, before and after the administration of VA molecules, were calculated from corresponding V_rev_ using the Goldman–Hodgkin–Katz equation. Data are represented as mean ± SD of *n* = 3 independent experiments.

	P_Cl−_/P_K+_ (O)	P_Cl−_/P_K+_ (C)
apo – hVDAC1	1.31 ± 0.07	0.59 ± 0.03
hVDAC1 + VA‐D11	1.58 ± 0.04	1.11 ± 0.02
hVDAC1 + VA‐D10	1.67 ± 0.09	1.24 ± 0.04
hVDAC1 + VA‐C1	1.55 ± 0.06	1.15 ± 0.10
hVDAC1 + VA‐C4	1.56 ± 0.07	1.25 ± 0.10
hVDAC1 + VA‐C6	1.59 ± 0.10	1.26 ± 0.05

## Discussion

The enhancement of aerobic glycolysis and the increased glucose uptake that lead to the downregulation of oxidative phosphorylation are key strategies, while not unique [49], that allow tumor cells to achieve proliferation advantages. VDAC1, as the master regulator of mitochondrial energy metabolism, actively sustains cancer energy reprogramming and represents an emerging target for antitumor therapeutics [37, 50, 51, 52, 53, 54]. Notwithstanding, almost all the potential drugs developed so far to act via VDAC are unsuitable for clinical trials because of high toxicity due to the lack of both specificity for the mitochondrial porin/s and common structural motifs [25]. Small molecules *ad hoc* designed against the NADH‐binding pocket of VDAC1 have recently proven to be a turning point in the selective killing of malignant cells [36, 37, 38, 39, 40]. Based on the promising antiproliferative effects registered in cultured cancer cells and in living organoids, it has been suggested these drugs may function as precise “VDAC openers” that counteract the partial suppression of mitochondrial metabolism by increasing the flux of metabolites across the channel and the cytosolic ATP/ADP ratio, eventually contributing to inhibition of glycolysis [38, 55]. To address this hypothesis, we explored the effects of five novel VDAC‐targeting small molecules, previously selected through an *in silico*‐to‐*in vitro* approach [40], on the electrophysiological properties of human VDAC isoform 1. Once added to both sides of a planar lipid membrane containing hVDAC1, none of the tested drugs affected the high‐conductance open state of the channel that lasted rather stable throughout the recording time. Vice versa, the voltage dependence of the pore was significantly impaired. The current response to triangular *voltage ramps* applied to a single *h*VDAC1 channel demonstrated that most of the molecules delayed pore closure up to potentials higher than *±30* mV. The transition from the unique open to the multiple closed states was not sharp, as pretty much all drugs caused hVDAC1 to continuously fluctuate between two or more conductance levels. Hereupon, the probability of the single channel being open calculated as a function of the applied transmembrane potential was statistically increased in the presence of the tested molecules. The VA drugs, however, did not uniformly affect the voltage dependence of the channel. This remains an unresolved issue that requires further investigations, considering that all VA molecules have comparable affinity to the channel [40]. One possible explanation could be that, aside from their common three‐rings architecture, differences in their atomic substituents influence their kinetic behavior and binding dynamics. Changes in the gating behavior of hVDAC1 suggest these compounds exclusively interfere with the voltage response, as they reduced the frequency of closure and forced the channel to stay open even at high potentials, without perturbing the maximum conductance state at low voltages. It is quite clear that, despite sharing part of the β‐NADH‐binding pocket [40, 41], the five VA molecules modulate hVDAC1 electrophysiology differently from β‐NADH itself. Contrary to the findings of Colombini and colleagues [36], our results show indeed that β‐NADH reduces hVDAC1 conductance already at ±10 mV applied and freezes the channel in a persistent low‐conductance state. The rationale behind these discrepancies could be related to the fact that in [36] authors exploited a human VDAC1 in which the first 10 N‐terminal amino acids were replaced with the first nine residues of yeast homologous. Recently, Hiller and coworkers attributed the drop in pore conductance triggered by β‐NADH‐binding exclusively to the steric blockage of the anion flux with a mechanism separated from the gating process [41]. In this regard, it is important to emphasize that, beyond the substantial difference in molecular mass, residues N238 and, in particular, K236 – responsible for the establishment of cation‐π interaction with the L‐ring of VDAC‐targeted molecules – are not essential for the binding of β‐NADH [40]. Noteworthy, K236 has been reported to be involved in hydrogen bonding with the backbone of residue F18 located in the N‐terminal moiety [16], so it is conceivable that our drugs interfere with the movement of the α‐helix required to modulate the channel upon voltage raising, as in [41]. K236 and N238 have been identified as key anchoring residues also in the interaction of the antiarrhythmic compound efsevin with the inner wall of zebrafish VDAC2 (zVDAC2) β‐barrel [56]. However, unlike VA molecules, efsevin binding promoted channel closure rather than hindered it, probably due to both differences in the *a*mino acid sequences surrounding the recognition site in zVDAC2 compared to hVDAC1 and in the chemical structure of the drugs. No data referring to the efsevin effect on tumor cells are available. If the results described so far reflect the initial hypothesis (i.e., VDAC‐targeted molecules effectively maintain hVDAC1 in an open configuration, opposing voltage‐induced closure), ion selectivity measurements demonstrated, for the first time, VDAC antagonists exert their antiproliferative activity on cancer cells by “reawakening” the mitochondrial metabolism through increased flux of anionic metabolites into and out of mitochondria. Accordingly, VA‐D10, VA‐D11, VA‐C1, VA‐C4, and VA‐C6 enhanced anion permeability in the open state, markedly suppressing the pore preference for positively charged ions in the closed state as well. Quite the opposite, β‐NADH maintained the pore in a voltage‐insensitive state endowed with a more pronounced cation selectivity than apo‐hVDAC1. These latest data are particularly relevant in light of the fact that VDAC1, along with the inositol 1,4,5‐trisphosphate receptor (IP3R) and the glucose‐regulated protein 75 (Grp75), mediates mitochondrial Ca^2+^ uptake at the mitochondria‐ER contact sites (MERCs) [5, 57, 58, 59, 60, 61], which has been proved to be essential for maintaining basal levels of OXPHOS and ATP production not only in normal cells but also in many tumor cell lines [62, 63, 64]. Strikingly, shutdown of constitutive ER‐to‐mitochondrial Ca^2+^ transfer through the inhibition of IP3R activity has been correlated to a bioenergetic crisis that culminates in the activation of AMPK‐dependent and mTOR (mammalian target of rapamycin)‐independent autophagy both in primary human fibroblasts and in tumorigenic breast and prostate cell lines [64, 65, 66]. Though, whereas non‐tumorigenic cells simply slowed proliferation by restricting their entry into the cell cycle, cancer cells exhibited an extremely susceptivity to Ca^2+^ as they did not arrest cell cycle in response to AMPK activation and underwent necrosis during cytokinesis. It is tempting to speculate that reducing permeation to cations could therefore be the keystone mechanism underlying the selective lethality of VA molecules to cancer cells [40]. Beyond this conclusion, it must be considered that VDAC opening is a double‐edged sword: while it facilitates the transport of anionic metabolites, it simultaneously boosts ROS production. This, in turn, drives oxidative stress‐induced mitochondrial dysfunction and bioenergetic failure, ultimately leading to cell death [67, 68]. According to Maldonado and coworkers indeed, erastin and erastin‐like compounds trigger ferroptosis in malignant cells by reversing the tubulin‐blocked state of VDAC that limits the entry of respiratory substrates and decreases Δ*ψ*_m_. This may explain why 48‐h treatment with VA molecules, as reported in [40], selectively impaired mitochondrial respiration in cancer cells, which overexpress VDAC1, but had no effect on normal cells [40, 69]: it is plausible that the chronic accumulation of ROS due to the initial stimulation of mitochondrial bioenergetics triggered by VDAC opening leads in the end to the mitochondrial collapse. In line with these considerations, the biophysical approach exploited in this work improved the comprehension of the antitumorigenic activity of VDAC‐targeted compounds, most likely ascribable to the combination of diverse cellular alterations driven by non‐compliant VDAC's gating concurring in cancer cell death. In‐depth studies are still required to fully characterize the metabolic changes evoked by the VA molecules and to optimize their chemical structure in view of a future application as cancer therapeutics.

## Conclusion

In conclusion, our findings propose a potential mechanism for the selective antitumor activity of VDAC‐targeting small molecules. By stabilizing VDAC1 in its open state and preventing voltage‐dependent closure, these compounds act as “VDAC openers” that enhance anion permeability and concurrently limit cation flux. This dual modulation may both reactivate mitochondrial metabolism in cancer cells – where the progressive buildup of oxidative stress can ultimately damage mitochondria and promote cell death – and reduce VDAC1 cation permeability, conceivably impairing the ER‐to‐mitochondria Ca^2+^ flux required to sustain OXPHOS. Nevertheless, additional studies are required to further define structure–activity relationships and optimize the pharmacological properties of these compounds. Overall, our study reinforces the rationale for targeting VDAC1 as a strategy to compromise tumor bioenergetics and proliferation.

## Materials and methods

### Heterologous expression and purification of recombinant human VDAC1

Human, recombinant 6xHis‐tagged VDAC1 was produced by heterologous expression in BL21 (DE3) *E. coli* strain and extracted from inclusion bodies, as reported in [70]. Briefly, isolated inclusion bodies were resuspended in Lysis buffer (50 mm Tris/HCl, 2 mm EDTA, 20% sucrose, pH 8.0). After sonication and centrifugation, inclusion bodies were resuspended in Wash buffer (20 mm Tris/HCl, 300 mm NaCl, 2 mm CaCl_2_, pH 8.0) and solubilized in Equilibration buffer A (20 mm Tris/HCl, 300 mm NaCl, 8 m Urea, pH 8.0) for 3 h at 4 °C under constant stirring. Recombinant human VDAC1 was purified by affinity chromatography with the HisTrap HF 5 mL affinity column (Cytiva, Marlborough, MA, USA). The column was washed with 5 column volumes (CV) of Equilibration buffer A containing 30 mm imidazole and the protein was eluted with 8 CV of Equilibration buffer A containing 150 mm imidazole.

### *In vitro* refolding and size‐exclusion chromatography

Refolding of hVDAC1 was carried out by gradual dropwise dilution of the protein solution at a rate of 100 μL·min into refolding buffer (25 mm Tris/HCl, 300 mm NaCl, 1 mm EDTA, 5 mm DTT, 1% (w/v) LDAO, pH 8.0), using a pellet‐to‐buffer ratio of 1 : 20 (w/v) [70]. The refolding process was performed at 4 °C for 3 h. The protein was then dialyzed overnight at 4 °C against dialysis buffer composed of 25 mm Tris/HCl and 300 mm NaCl at pH 8.0. To decrease the detergent concentration, the dialyzed protein was loaded onto a 5 mL HisTrap HP affinity column, as reported in [40, 70]. Finally, the sample was subjected to size‐exclusion chromatography on a Superdex 200 Increase 10/300 column (Cytiva). The monomeric fraction was collected following elution with SEC buffer (20 mm Tris/HCl, 50 mm NaCl, 0.1% (w/v) LDAO, pH 8.0). The monomeric state of hVDAC1 was confirmed by SDS/PAGE.

### Analysis of hVDAC1 conductance

The reconstitution of hVDAC1 was carried out as previously described [10, 13, 71, 72, 73, 74] using a PLB setup. Specifically, asolectin (20 mg·mL^−1^ in *n*‐decane) was applied to a 200 μm hole in a Delrin cuvette to form the bilayer. Experimental consistency was ensured by selecting only membranes with a capacitance of 90–120 pF. After establishing the bilayer, 40 ng of refolded protein was added to the *cis* side in a symmetrical 1 m KCl buffer (10 mm HEPES, pH 7.0) to promote channel insertion. Current signals were captured with a Bilayer Clamp amplifier (Warner Instruments), filtered at 300 Hz, and digitized at 100 μs/point. Analysis was performed using pClamp 10 software. To evaluate the impact of β‐NADH, DMSO, and specific VA molecules (VA‐D11, VA‐D10, VA‐C1, VA‐C4, and VA‐C6), these substances were introduced to both the *cis* and *trans* sides of the chambers at concentrations of 15 μm, 0.6%, and 20 μm, respectively. Each experiment was performed at least in triplicate to ensure reproducibility.

### Evaluation of hVDAC1 voltage dependence

The voltage dependence of hVDAC1 was measured in symmetrical KCl solution by applying 10 mHz triangular voltage waves of ±50 mV for 100 s. Plots of the average conductance of hVDAC1 as a function of voltage were obtained as reported in [75]. The relative conductance was calculated as G/G_max_, where G denotes the average conductance at a given voltage and G_max_ represents the maximum conductance of the channel, corresponding to the fully open state, and is calculated from the slope of the I–V relationship in the high‐conductance state. The percentage decrease in hVDAC1 voltage dependence upon VA molecules was measured by acquiring the Area Under Curve (AUC) from G/G_max_ plots, as described in [75]. Accordingly, the AUC values of hVDAC1 treated with each of the tested compounds were subtracted from the AUC value of the untreated protein. The open probability (P_open_) as a function of the applied voltage was calculated from single‐channel recordings following application of triangular voltage waves of ±50 mV as previously performed in [76]. V_0_ was calculated by fitting the P_open_ versus the V plot with the Boltzmann equation as reported in [77]. The kinetic analysis for hVDAC1 in the presence or absence of the VA drugs was obtained from single‐channel recording experiments by applying voltage steps of ±10 mV over a range of ±30–50 mV for 40 s. The amplitude values obtained from the current traces were plotted as a function of the number of events using clampfit 9.0 (Molecular Devices, San Jose, CA, USA), as previously reported [10, 56, 78]. Histograms of the dwell time values in the open state were generated from the current traces and plotted as a function of the normalized number of events, also using clampfit 9.0, and subsequently analyzed with the same software. Dwell time values under each experimental condition were plotted as a function of the applied voltage, and linear regression analyses were performed as reported in [41, 79, 80]. For each condition, at least *n* = 3 independent experiments were carried out. Graphs were generated using graphpad prism 9 software (GraphPad Software, San Diego, CA, USA).

### Ion selectivity measurements

Ion selectivity was estimated from the reversal potential ($V_{r}$) obtained in asymmetric ionic conditions using the Goldman–Hodgkin–Katz (GHK) equation, as described in [13]. Accordingly, channel insertion was first achieved in symmetrical 1 m KCl solution. After incorporation of at least one channel, the *cis* side was perfused with approximately 10 chamber volumes of 0.1 m KCl to establish a 0.1 m *cis*/1 m *trans* KCl gradient. A triangular voltage wave (±50 mV, 10 mHz; 100 s period) was then applied to determine the reversal potential from the current–voltage relationship. The $V_{r}$ values were subsequently used in the GHK equation to calculate the permeability ratio of anions (P_Cl−_) over cations (P_K+_). At least *n* = 3 independent experiments were performed for each analysis.

### Statistical analysis

Data are expressed as a mean ± SD and statistically analyzed by Student *t*‐test or one‐way ANOVA followed by Tukey's test using prism software (version 9.0, GraphPad Software, Boston, MA, USA). The values of * *P* < 0.05, ** *P* < 0.01 and *** *P* < 0.0 01 were taken as significant.

## Conflict of interest

The authors declare that they have no actual or potential conflict of interest including any financial, personal or other relationships with other people or organizations within 3 years of beginning the submitted work that could inappropriately influence or be perceived to influence their work.

## Author contributions

SCN performed hVDAC1 expression and purification, chromatography, electrophysiological experiments, discussed the data, drew the pictures and contributed to the manuscript preparation. GB contributed to VDAC1 purification and SAMC contributed to electrophysiology experiments. CA and ML analyzed and interpreted the data, contributed to the manuscript preparation and revised the manuscript. SR and VDP conceived the idea, conceptualized, designed and supervised the study, provided the financial support (VDP), wrote the original draft, revised and finalized the manuscript.

## Data Availability

The data that support the findings of this study are available from the corresponding author [simona.reina@unict.it; vito.depinto@unict.it] upon reasonable request.
